# 

**Published:** 1902-09

**Authors:** 


### PERSONALS.

State Veterinarian Pearson, of Pennsylvania, addressed the dairymen's gathering at Castle Rocks Park, Chester County, in August.

Dr. William Gall, of Matawan, N. J., made a trip to Europe in the latter part of the summer season.

Dr. J. H. Oyler, of Harrisburg, Pa., is the owner of the fast trotting gelding "Blizzard." He was the winner in a $300-purse at Lancaster, in August.

Dr. E. Brainerd, of Memphis, Mo., spent his vacation in Utah and Colorado, in the early days of August.

State Veterinarian Pearson made a flying trip to Quebec, Canada, in August, visiting Montreal, Toronto, Guelph, and many other points.

Dr. Emil Knight, of Rochester, N. Y., spent many of the days of August in Atlantic City, N. J., listening to what the wild waves were saying.

Dr. J. C. McNeil, city veterinarian of Pittsburg, Pa., spent two weeks in Altantic City and at Manhattan Beach in August.

Dr. Olaf Schwartzkoff, of the United States army, recently stationed in the Philippine Islands, has returned to the United States with the Third Cavalry, and will be stationed at Cinnabar, Wyoming, near the Yellowstone Park.

Dr. J. M. Wright, of the firm of Wright and Merillat, of Chicago, hied himself to the woods of Wisconsin for a period of ten days in August.

Dr. A. S. Alexander, of Evanston, Ill., contributed a very interesting article to a recent issue of the *Weekly Live-stock Report*, entitled "Preventing Hog Cholera."

Dr. Nelson P. Hinkley, formerly of Atlanta, Ga., has returned to his old home, Buffalo, N. Y., owing to the impaired health of his wife in the South. He is rapidly regaining his old clientele.

Dr. John R. Mohler, of the Bureau of Animal Industry, made a trip recently through the South Dakota ranches.

Dr. R. F. Forrest, of Uxbridge, Ontario, is a breeder and admirer of the trotter.

Dr. W. Ratcliffe and family, of Waynesburg, Pa., spent some four months this summer travelling through England.

Dr. S. S. Cole, of Vineland, N. J., was the defendant in a peculiar suit for alleged injuries done by a bull-dog. His under-shot mouth proved that he could not bite.

Dr. I. Johnson, of Peterborough, Canada, takes delight in handling green trotters and preparing them for the race track.

Dr. Thomas MacColey, veterinarian, of Carnegie, Pa., is running an independent candidacy for the office of burgess of his adopted city. He has framed his own platform, and will appear on a citizens' ticket, ignoring the recognition of both old parties.

Dr. D. E. Salmon, Chief of the Bureau of Animal Industry, will visit England shortly, to inspect the methods of shipment of meats in refrigerator ships.

Dr. S. F. Rose, of Waynesburg, Pa., was killed during the summer by being struck with a bottle.

Dr. D. Eugene Block, Secretary of the Virginia Field and Sporting Club and ex-Veterinary Surgeon of the United States army, is now engaged in the training and sale of saddle horses and those suitable for hunting and jumping purposes.

Drs. R. F. Eagle, D. G. Painter, H. Timmerman, F. W. Hopkins, and U. G. Houck were among the East St. Louisians in attendance at the eleventh annual meeting of the Missouri State Veterinary Association, St. Louis, August 18th and 19th.

Dr. W. A. Boucher, of Salina, Kas., has located at Fullerton, Cal., where he will continue the practice of his profession.

Dr. T. M. Doram, graduate of the McKillip Veterinary College, has located at Danville, Ky.

Veterinary Surgeon Howes, of the English army, is severely criticised because of his close relations with Captain Smith, who is now under arrest for connivance and corruption in the purchase of mules for the South African army while stationed at New Orleans, La.

Dr. Otto G. Noack is Secretary of the Road Drivers' Association, of Reading, Pa. The organization is agitating the question of a speedway for Reading road drivers.

The Chicago Horseshow Association has selected as its veterinarians Dr. H. M. McKillip, chief veterinarian, and as staff veterinarians Dr. Frank Allen, N. E. Nettleton, and Gerald E. Griffin. This makes a strong array of veterinary service.

Dr. Levi Johnson, of Portland, Ore., is attending a post-graduate conrse at the McKillip Veterinary College.

Dr. C. M. Noble, of Houston, Texas, has been appointed food inspector for that city at a salary of $1200 per year.

Dr. W. Harry Lynch, of Manchester, N. H., has become the assistant of Dr. J. H. Ferster, of West Eleventh Street, New York City, N. Y.

Dr. O. A. Stingley, of Kansas City, has been appointed an assistant inspector in the Bureau of Animal Industry, and ordered to Chicago.

---

### NOTES.

South Carolina has recently issued her Veterinary Sanitary Control Regulations in pamphlet form. As these rules and statutory provisions are promulgated through the Clemson Agricultural College, to which is attached the State Experiment Station, and as the State Veterinarian is an officer of the college and Experiment Station, there is an additional interest to their police measures.

Quarter evil : Dr. Marra has treated two cases with tachyol, a new antispetic discovered by Prof. Pasteur and used with success in Milan Veterinary College. Marra made ninety injections in the swellings on two calves, commencing in the periphery and working to the centre, the solution being 1 to 500. Each had a dose of hay tea, with 100 grammes of alcohol. The temperatures in the morning before the first injection were 41.4° C., 41.5° C.; evening, 39.5° C. Next day, 38.4° C. and 38° C. The swelling was stationary for forty-eight hours; then the animals recovered their spirits and began to eat. "In five days," wrote the owner, "they were all right."—*Policlinica.*

The first dog hospital in Moscow has just been founded by an association of women of that place.

